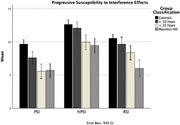# Exploring Verbal Memory Deficits in Huntington's Disease: Novel Insights from the LASSI‐L

**DOI:** 10.1002/alz70857_098429

**Published:** 2025-12-24

**Authors:** Luis A. Sierra

**Affiliations:** ^1^ William James College, Newton, MA, USA; Beth Israel Deaconess Medical Center, Boston, MA, USA

## Abstract

**Background:**

Huntington's disease (HD) is marked by progressive cognitive decline, with early deficits often presenting long before motor symptoms. These changes are subtle yet profoundly impactful, underscoring the need for sensitive tools like the Loewenstein‐Acevedo Scales for Semantic Interference and Learning (LASSI‐L). The LASSI‐L evaluates susceptibility to semantic interference, an area of cognitive functioning likely influenced by HD pathology. This study explores how specific verbal memory deficits progress across different stages of HD, providing insights into disease staging.

**Method:**

A total of 151 participants (89 HD, 62 healthy controls) were recruited across three sites. The HD group was further classified into >10 years from manifest HD, <10 years from manifest HD, and manifest HD, using PIN scores and Total Motor Score (TMS). Performance on the LASSI‐L—including Proactive Semantic Interference (PSI), Failure to Recover from PSI (*fr*PSI), and Retroactive Semantic Interference (RSI)—was compared across groups using ANCOVAs adjusted for age, followed by post‐hoc pairwise comparisons with Bonferroni correction.

**Result:**

A sequential pattern of verbal memory deficits emerged. PSI deficits were evident >10 years before Manifest HD, reflecting early cognitive vulnerabilities. As the disease progressed, *fr*PSI deficits appeared <10 years from manifest HD, with further declines seen in RSI during the manifest stage. Notably, 98% of HD participants displayed impairments, with 88% following a clear progression from PSI to *fr*PSI to RSI.

**Conclusion:**

The LASSI‐L provides a nuanced lens to examine cognitive changes in HD, capturing the subtle yet progressive nature of semantic interference deficits. These findings suggest that the LASSI‐L is a valuable tool not only for detecting early cognitive changes but also for refining disease staging. Its ability to delineate the progression of verbal memory impairments highlights its potential to advance our understanding of HD‐related cognitive decline.